# Infections and hypogammaglobulinemia in myasthenia gravis patients receiving low-dose rituximab: a real-world cohort study

**DOI:** 10.3389/fimmu.2026.1907574

**Published:** 2026-08-28

**Authors:** Rongjing Guo, Yifan Zhang, Ting Gao, Zhe Ruan, Chao Sun, Sijia Hao, Yonglan Tang, Qingqing Wang, Xiaoxi Huang, Xiangqi Cao, Zhuyi Li, Danni Liu, Yu Liu, Ting Chang

**Affiliations:** Department of Neurology, Tangdu Hospital, The Fourth Military Medical University, Xi’an, Shaanxi, China

**Keywords:** prednisone, hypogammaglobulinemia, infection, myasthenia gravis, rituximab

## Abstract

**Aims:**

We investigated the incidence of infections and hypogammaglobulinemia (HGG) in patients with myasthenia gravis (MG) receiving long-term low-dose rituximab (RTX) maintenance therapy and identified related risk factors.

**Methods:**

This single-center retrospective cohort study enrolled MG patients treated with low-dose RTX maintenance. Cox proportional hazards regression was performed to analyze risk factors for infections and HGG.

**Results:**

A total of 186 patients were included. The median age at RTX initiation was 52.50 years, and the median follow-up was 2.41 years. Infections occurred in 29 patients (15.59%), with a median time to first infection of 170.37 days, mostly during the first cycle. The 1- and 2-year infection rates were 13.41 (95% confidence interval [CI], 8.47–20.28) and 9.20 (95% CI, 6.06–13.36) per 100 person-years. Severe infections developed in 21 patients (11.29%). The most prevalent infection was lower respiratory tract infection (LRTI), with 19 episodes (61.29%). HGG was present in 51 patients (27.42%). HGG was an independent risk factor for infection (HR = 5.53, P<0.001). Higher baseline prednisone dose (HR = 1.25, P = 0.035) and HGG (HR = 7.50, P<0.001) were significantly correlated with increased risk of severe LRTIs.

**Conclusion:**

In MG patients receiving long-term low-dose RTX maintenance, the overall infection incidence is relatively low, with LRTIs being the most frequent. HGG is a major independent risk factor for infections and severe LRTIs, while higher baseline prednisone doses were associated with severe LRTIs.

## Introduction

Myasthenia gravis (MG) is an autoimmune disorder that affects the neuromuscular junction and is characterized by impaired neuromuscular transmission. It is primarily mediated by pathogenic autoantibodies against the acetylcholine receptor (AChR), muscle-specific receptor tyrosine kinase (MuSK), and low-density lipoprotein receptor–related protein 4 (LRP4) (1). These autoantibodies disrupt neuromuscular transmission by blocking acetylcholine interaction, reducing receptor density, and activating complement cascades, resulting in muscle weakness ranging from isolated ocular involvement to life-threatening respiratory muscle impairment (2).

Myasthenia gravis (MG) is a B-cell-mediated autoimmune disease (3). Based on this pathogenic mechanism, B-cell-depleting therapeutic techniques have emerged as important strategies for treating MG. Currently used agents in clinical practice that target B cells include rituximab (RTX) (4), inebilizumab (5), and ofatumumab (6). RTX is a chimeric human-mouse IgG1 monoclonal antibody directed against the CD20 antigen expressed on B lymphocytes. Several clinical trials and real-world studies have demonstrated that RTX is effective for managing patients with refractory or newly diagnosed MG (7–9). In a double-blind, placebo-controlled trial conducted in Sweden in 2022 (4), intravenous administration of 500 mg of RTX significantly improved clinical scores at 16 weeks compared to placebo, while also reducing prednisone requirements and the need for rescue therapy.

Although RTX is an off-label therapy for MG, it has become a widely used treatment option worldwide because it is efficacious and cost-effective. Consequently, researchers have paid close attention to its safety profile. Reported adverse events include infections, infusion-related reactions, hypogammaglobulinemia (HGG), and, rarely, progressive multifocal leukoencephalopathy (PML) (10–12). Among patients with autoimmune diseases treated with RTX, the rates of serious infections, opportunistic infections, and mortality were found to be 28.1%, 7.8%, and 4.7%, respectively (13). Once infection occurs, it may gravely threaten patient survival and complicate subsequent treatment.

Systematic data on the risk of infection associated with RTX treatment in MG are limited (14–16). Infections can significantly precipitate myasthenic crisis (17), while respiratory muscle paralysis and dysphagia during crisis may further exacerbate the infection, complicating clinical management. This bidirectional relationship highlights the clinical importance of preventing and controlling infections during RTX therapy. Therefore, we conducted this retrospective cohort study using real-world data from a large tertiary medical center in China to systematically characterize the incidence, clinical features, and risk factors of infections in MG patients receiving 500 mg of RTX every six months. We also evaluated the incidence of HGG, aiming to provide robust evidence for individualized prevention of infections and management strategies in clinical practice.

## Materials and methods

### Study design and population

We conducted a single-center retrospective cohort study at the Second Ward of Neurology, Tangdu Hospital of the Fourth Military Medical University. The participants were patients with MG who received RTX treatment between January 2019 and January 2025. All decisions regarding RTX treatment were made by the attending neurologists responsible for taking care of the patients.

**Inclusion criteria**: (1) patients who received 500 mg of RTX every 6 months; (2) those who were ≥18 years of age; (3) patients who had normal baseline levels of C-reactive protein, B-cell counts, and immunoglobulins, T cell spot test for tuberculosis (T-SPOT), before the first RTX infusion; and (4) those who underwent scheduled follow-up assessments at one to three, and six months after each infusion.

**Exclusion criteria**: (1) concomitant hematologic malignancies; (2) received non-fixed RTX doses; (3) irregular treatment intervals; or (4) a follow-up duration of less than three months without experiencing any infection.

**Figure 1:**
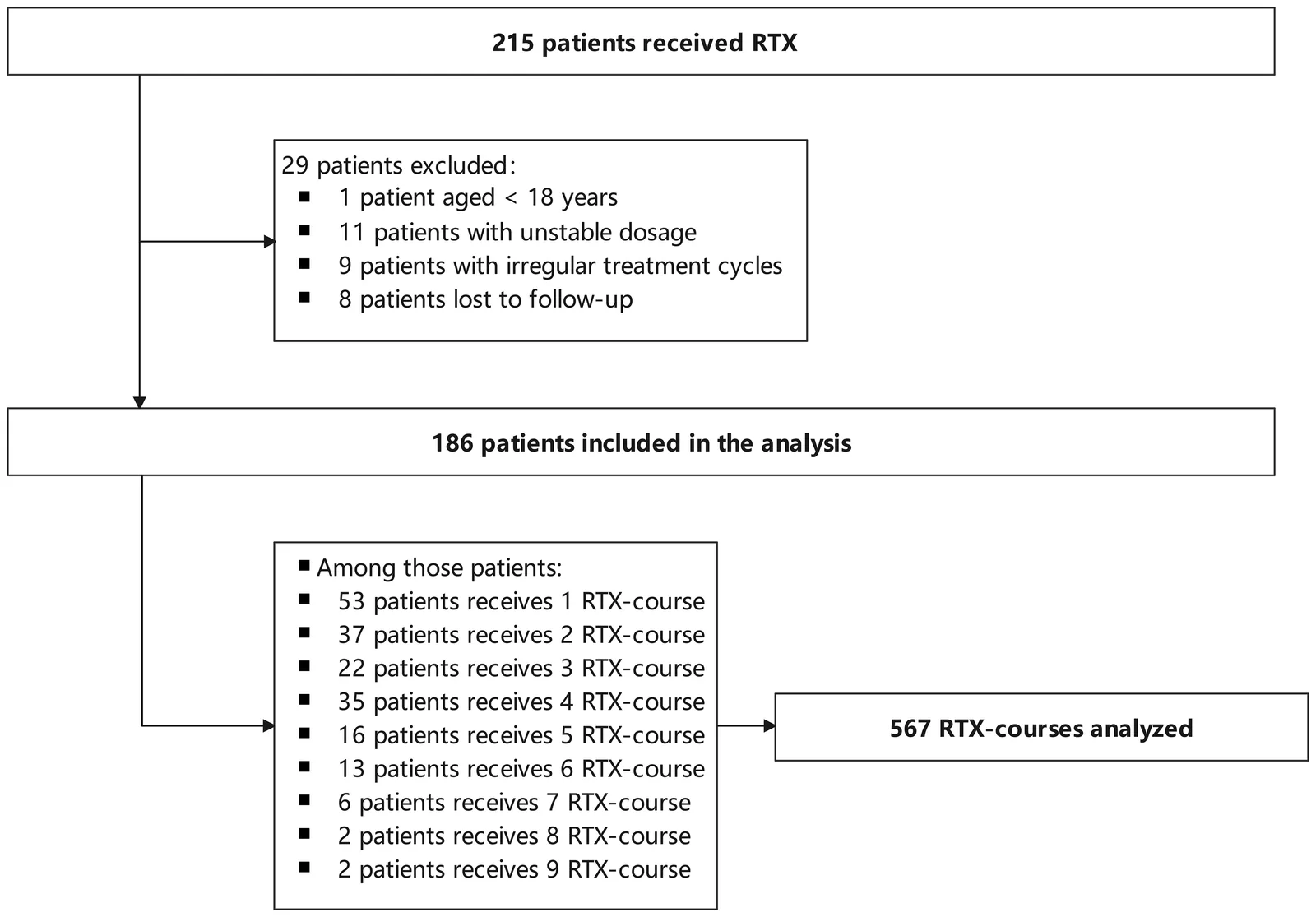
Flowchart of patient selection. (RTX: rituximab).

A total of 215 MG patients initiated RTX therapy during the study period. Among them, 29 patients were excluded for the following reasons: age <18 years (n = 1), a non-fixed dose of RTX (n = 11), irregular treatment intervals (n = 9), or loss to follow-up (n = 8). In the final analysis, 186 patients were included, corresponding to 567 RTX treatment cycles. Specifically, 53, 37, 22, 35, 16, 13, 6, 2, and 2 patients received 1–9 RTX treatment cycles, respectively. The patient selection process is illustrated in **Figure 1**.

### Data collection

**Baseline data** (collected before the first RTX infusion) included: (1) Demographic characteristics: age, sex, smoking history, and alcohol consumption; (2) Disease-related characteristics: disease duration (MG diagnosis to the first infusion of RTX), MG antibody subtype, Myasthenia Gravis Foundation of America (MGFA) clinical classification, Myasthenia Gravis–Activities of Daily Living (MG-ADL) score, and Quantitative Myasthenia Gravis (QMG) score, as well as the presence of bulbar or respiratory symptoms; (3) Comorbidities: diabetes mellitus, hypertension, cardiovascular or cerebrovascular disease, thyroid disorders, and other autoimmune diseases; (4) Laboratory parameters before administering RTX therapy: serum immunoglobulin levels (IgG, IgM, and IgA) and peripheral blood B-cell counts (percentage of CD19^+^/CD27^+^ cells among lymphocytes, CD19^+^ B-cell count, and CD20^+^ B-cell count); (5) Concomitant medications within 3 months before the initiation of RTX: pyridostigmine, prednisone, and non-steroidal immunosuppressants, along with their corresponding dosages. All doses of prednisone were converted into prednisone equivalents.

**Follow-up data** included: (1) Number of RTX treatment cycles and duration of follow-up; (2) occurrence of infections: the date of onset, infection type, severity, causative pathogens, treatment regimen, and prognosis; and (3) serum IgG, IgM, and IgA levels at each follow-up.

### Outcomes definition

#### Infection grading

All infections were diagnosed comprehensively according to unified diagnostic criteria incorporating clinical manifestations, laboratory examinations, microbiological evidence (bacterial culture or metagenomic next-generation sequencing), and imaging findings. All infectious cases were reviewed independently by two researchers in strict accordance with predefined criteria and complete medical records. This study included both nosocomial and community-acquired infectious cases. In patients complicated with pneumonia in this study, no definite aspiration episodes or acute hypoxic attacks were documented prior to disease onset. Pulmonary lesions were not limited to the bilateral lower lobes, and aspiration pneumonia was therefore excluded. All infection events were graded based on the Common Terminology Criteria for Adverse Events (CTCAE), version 5.0 (10). Grade 1: Asymptomatic or mild and requiring no intervention; Grade 2: Moderate infection, requiring local or noninvasive intervention; Grade 3: Severe infection, requiring intravenous antibiotics, antifungal, or antiviral therapy, or interventional radiologic or surgical procedures; Grade 4: Life-threatening infection (e.g., septic shock, hypotension, metabolic acidosis, tissue necrosis); Grade 5: Fatal infection: Severe infections were defined as Grade 3 or higher infections.

#### Hypogammaglobulinemia

Defined as reported in another study (18), as serum IgG, IgM, or IgA levels below the lower limit of normal. Low IgG was defined as <7 g/L, low IgM was defined as <0.4 g/L, and low IgA was defined as <0.7 g/L. Based on IgG levels, HGG was further classified as mild (5–7 g/L), moderate (3–5 g/L), or severe (<3 g/L).

### Statistical analysis

All statistical analyses were performed using R software (version 4.4.1). The normality of continuous variables was assessed using the Shapiro-Wilk test. Continuous variables that followed a normal distribution were presented as the mean ± standard deviation (SD), whereas continuous variables that did not follow a normal distribution were expressed as the median (interquartile range [IQR]). Categorical variables were presented as a number (percentage). Kaplan-Meier survival curves were constructed to calculate the cumulative incidence of infectious episodes and incident hypogammaglobulinemia (HGG). Cox proportional hazards regression models were adopted to explore risk correlates for predefined clinical endpoints. Schoenfeld residual tests confirmed that all covariates satisfied the proportional hazards assumption (P > 0.05). Collinearity among covariates was assessed using the variance inflation factor (VIF). All included variables had VIF values less than 3, indicating no obvious multicollinearity. Incident HGG occurring during follow-up was incorporated as a time-dependent covariate in primary regression models. To mitigate overfitting given the limited number of outcome events, parsimonious multivariate models were built by retaining predictors exhibiting P < 0.10 in univariate Cox regression alongside clinically critical confounders. All regression outputs were expressed as hazard ratios (HR) with corresponding 95% confidence intervals (CIs). Notably, the HR for prednisone reflected the relative risk increment associated with each 5 mg daily increase in equivalent prednisone dose.

One- and two-year infection incidence rates per 100 person-years were computed as (number of infectious events/total cumulative person-years) ×100. Poisson approximation was applied to calculate the 95% CIs for these incidence rates. All statistical tests were two-sided, and a threshold of P < 0.05 was designated to denote statistical significance.

## Results

### Patient characteristics

A total of 186 patients were included in the final analysis. The median age at initiation of RTX therapy was 52.50 years (IQR: 37.00–64.00), and 104 patients (55.91%) were female. The median disease duration was 24.50 months (IQR: 9.00–63.00). Anti-AChR and MuSK antibodies were positive in 135 (72.58%) and 28 (15.05%) patients, respectively. Comorbidities included diabetes mellitus (n=26, 13.98%), hypertension (n=50, 26.88%), cardiovascular/cerebrovascular disease (n=7, 3.76%), thyroid disorders (n=7, 3.76%), and other autoimmune diseases (n=5, 2.69%). In addition, 71 patients (38.17%) presented with isolated bulbar involvement, 15 (8.06%) with isolated respiratory involvement, and 47 (25.27%) with combined bulbar and respiratory involvement. The total follow-up time was 479.24 person-years, with a median follow-up duration of 2.41 years (IQR: 1.05–3.87). The median number of RTX treatment cycles per patient was 3.00 (range: 1.00–9.00). Among all patients, 185 individuals (99.46%) received at least one concomitant medication. Among these individuals, including pyridostigmine (n = 178, 95.70%), prednisone (n = 158, 84.95%), and non-steroidal immunosuppressants (n = 56, 30.11%). The most commonly used non-steroidal immunosuppressant was tacrolimus (n = 38, 20.43%), followed by azathioprine (n = 10, 5.38%) and mycophenolate mofetil (n = 7, 3.76%). The baseline characteristics of the study population are summarized in **Table 1**. The baseline characteristics of 29 patients excluded are summarized in **Supplementary Table 1**.

**Table 1:** Baseline characteristics of patients.

Variables	Total (n = 186)
Demographic characteristics	
Age, Y, Median (IQR)	52.50 (37.00, 64.00)
Female, n(%)	104/186 (55.91)
Smoking history	29/186 (15.59)
Alcohol history	13/186 (7.00)
MG disease characteristics	
Disease duration, Months, Median (IQR)	24.50 (9.00, 63.00)
Antibody type, n(%)	
AChR	135 (72.58)
MuSK	28 (15.05)
Other	4 (2.15)
Seronegative	19 (10.22)
MGFA stage, n(%)	
I	35 (18.82)
IIA	41 (22.04)
IIB	52 (27.96)
IIIA	16 (8.60)
IIIB	30 (16.13)
IVA	3 (1.61)
IVB	9 (4.84)
MG-ADL, Median (IQR)	5.50 (3.00, 8.00)
QMG, Median (IQR)	12.00 (7.25, 17.00)
Comorbidities	
Diabetes, n(%)	26/186 (13.98)
Hypertension, n(%)	50/186 (26.88)
CV/CVD, n(%)	7/186 (3.76)
Thyroid disease, n(%)	7/186 (3.76)
Other autoimmune diseases, n(%)	5/186 (2.69)
Bulbar/Respiratory involvement, n(%)	
Bulbar only	71/186 (38.17)
Respiratory only	15/186 (8.06)
Bulbar + Respiratory	47/186 (25.27)
B cell counts	
CD19+/CD27+B, Median (IQR)	2.91 (1.98, 5.26)
CD19+B, Median (IQR)	10.59 (7.25, 15.33)
CD20+B, Median (IQR)	10.32 (7.00, 15.21)
RTX treatment cycles, Median (Range)	3.00 (1.00 - 9.00)
Follow-up time, Years, Median (IQR)	2.41 (1.05, 3.87)
Concomitant medications, n(%)	185/186 (99.46)
Pyridostigmine, n(%)	178/186 (95.70)
Pyridostigmine daily dose, mg, Median (IQR)	210.00 (180.00, 352.50)
Prednisone, n(%)	158/186 (84.95)
Prednisone daily dose, mg, Median (IQR)	20.00 (20.00, 30.00)
Non-steroidal immunosuppressant, n(%)	56/186 (30.11)
Type of non-steroidal immunosuppressant, n(%)	
Efgartigimod	1 (0.54)
Azathioprine	10 (5.38)
Mycophenolate mofetil	7 (3.76)
Tacrolimus	38 (20.43)

IQR, interquartile range; AChR, acetylcholine receptor; MuSK, muscle-specific receptor tyrosine kinase; MGFA, Myasthenia Gravis Foundation of America Clinical Classification; MG-ADL, Myasthenia Gravis Activities of Daily Living; QMG, Quantitative Myasthenia Gravis; CV/CVD, cardiovascular/cerebrovascular disease; RTX, rituximab; Range, observed range.

### Incidence of infections and HGG after RTX therapy

**Figure 2:**
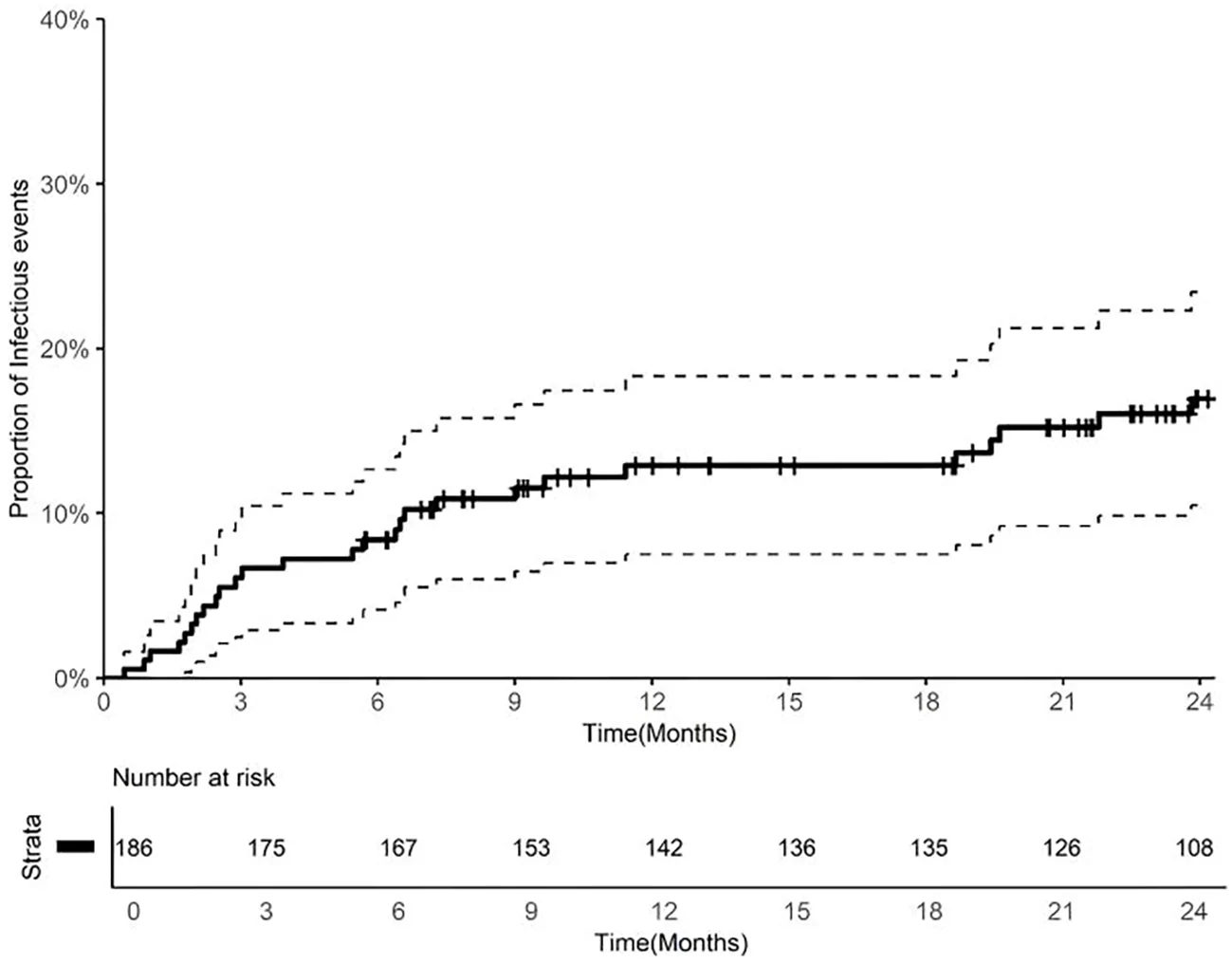
Time to first infection in patients after the initiation of RTX treatment.

After RTX therapy was initiated, 29 patients (15.59%) experienced 31 infectious episodes, of which 21 patients (11.29%) developed 23 severe infections (≥Grade 3) (**Table 2**). The median time to the first infection was 170.37 days (IQR: 65.60–342.63), and the median RTX treatment cycle at the time of infection was 1 (range: 1–6 cycles). **Figure 2** depicts the Kaplan-Meier curve for time to first infection following RTX initiation. The number of patients at risk at each time point was 186 at month 0, 175 at month 3, 167 at month 6, 153 at month 9, 142 at month 12, 136 at month 15, 135 at month 18, 126 at month 21, and 108 at month 24. Among all types of infections, lower respiratory tract infections (LRTIs) (N = 19) were the most frequent, accounting for 61.29% of all episodes (**Table 2**). The 1- and 2-year infection incidence rates were 13.41 (95% CI, 8.47-20.28) and 9.20 (95% CI, 6.06-13.36) per 100 person-years, respectively.

**Table 2:** Infectious events in MG patients receiving RTX.

Type of infection	No. of episodes (%)
Skin infection	2 (6.45)
Upper respiratory tract infection	5 (16.13)
Gastrointestinal infection	3 (9.68)
Lower respiratory tract infection	19 (61.29)
Urinary tract infection	1 (3.23)
Central nervous system infection	1 (3.23)
Total	31

Severe LRTIs occurred in 18 patients (9.68%). Among the 29 patients who developed infections, the causative pathogen was identified in 17 patients (58.62%). Multiple pathogens could be detected in a single patient. The pathogens included 9 episodes of bacteria, 8 episodes of fungi, and 6 episodes of viruses. Notably, Pneumocystis jirovecii pneumonia was diagnosed in 7 of the 21 patients with severe infections (33.33%). Information on all infectious episodes is provided in **Supplementary Table 2**. Regarding outcomes, 25 of the 29 infected patients (86.21%) showed clinical improvement, 3 patients (10.34%) died (2 due to myasthenic crisis and 1 due to pulmonary infection), and 1 patient (3.45%) had an unknown outcome.

**Figure 3:**
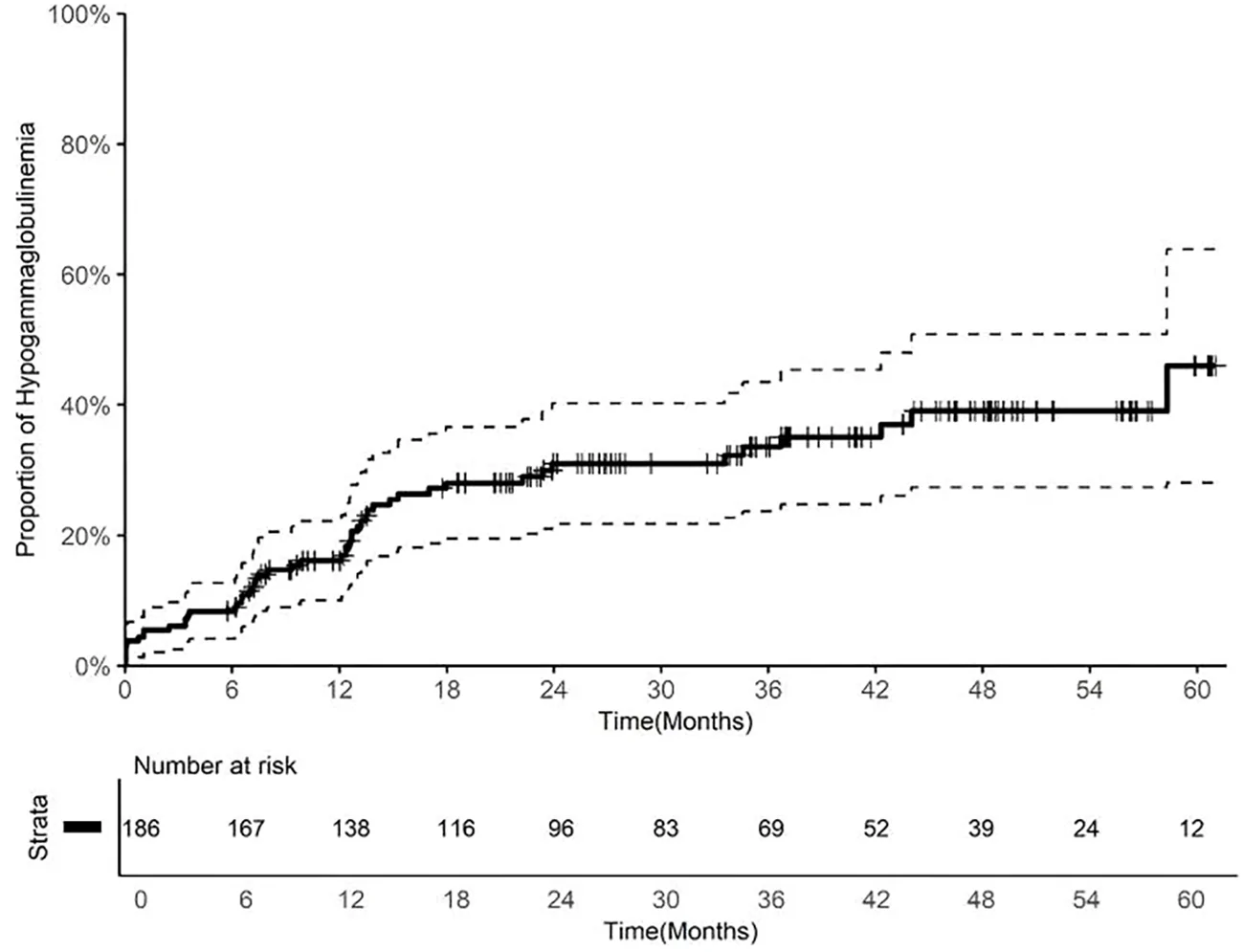
Time to HGG in patients after the initiation of RTX treatment.

During the follow-up period, HGG was detected in 51 patients (27.42%), with a median time to HGG onset of 218.36 days (IQR: 82.02–400.48). In the overall study population, low IgG levels were recorded in 18 patients (9.68%), low IgM levels were recorded in 37 patients (19.89%), and low IgA levels were recorded in six patients (3.23%). Among patients with low IgG levels, nine patients (50.00%) had mild HGG, whereas four (22.22%) and five patients (27.78%) had moderate and severe HGG, respectively. Additionally, among patients who developed HGG, eight patients (15.69%) showed reductions in two immunoglobulin classes, and one patient (1.96%) showed reductions across all three immunoglobulin classes (**Table 3**). Among 15 patients who developed infection following HGG diagnosis, the median interval from HGG diagnosis to subsequent infection was 65.00 days (IQR: 1.00–195.50). The cumulative incidence of HGG in MG patients receiving RTX is shown in **Figure 3**. The number of patients at risk at each predefined time point was 186 at month 0, 167 at month 6, 138 at month 12, 116 at month 18, 96 at month 24, 83 at month 30, 69 atmonth 36, 52 at month 42, 39 at month 48, 24 at month 54, and 12 at month 60.

**Table 3:** The distribution of HGG in MG patients receiving RTX.

Type of HGG	N(%) (proportion of HGG, N = 51)
IgG+IgM+IgA+	1 (1.96)
IgG+IgM+IgA-	4 (7.84)
IgG+IgM-IgA+	1 (1.96)
IgG-IgM+IgA+	3 (5.88)
IgG+IgM-IgA-	12 (23.53)
IgG-IgM+IgA-	29 (56.86)
IgG-IgM-IgA+	1 (1.96)

Note: “+” indicates decreased immunoglobulin levels, and “−” indicates normal levels. Low IgG, IgM, and IgA levels were defined as serum IgG <7 g/L, IgM <0.4 g/L, and IgA <0.7 g/L, respectively. For low IgG, mild event was defined as IgG 5–7 g/L, moderate event was defined as 3–5 g/L, and severe event was defined as <3 g/L.

### Risk factors for infection and HGG after RTX treatment

#### Risk factors for overall infections

Univariate Cox regression analysis showed that cardiovascular/cerebrovascular disease (HR: 3.35, 95% CI:1.01-11.08, P = 0.047), higher daily prednisone dose (HR: 1.19, 95% CI: 1.02-1.40, P = 0.030), and the development of HGG during follow-up (HR: 3.02, 95%CI: 1.46-6.25, P = 0.003) were significantly associated with an increased risk of overall infections. Multivariate Cox regression analysis confirmed that HGG was the only independent risk factor for overall infections (HR: 5.53, 95% CI: 2.42-12.60, P < 0.001) (**Table 4**).

**Table 4:** Multivariate cox regression analysis of the first infection.

Variables	No (n = 157)	Yes (n = 29)	HR (95% CI)	*P*
CV/CVD	4 (2.55)	3 (10.34)	2.91 (0.82 ~ 10.40)	0.099
Prednisone daily dose/5 mg	4.00 (4.00, 6.00)	6.00 (4.00, 8.00)	1.12 (0.95 ~ 1.33)	0.175
HGG (time-dependent covariate)	36 (22.93)	15 (51.72)	5.53 (2.42 ~ 12.60)	<0.001

CV/CVD, cardiovascular/cerebrovascular disease; HGG, hypogammaglobulinemia; HR, hazard ratio; CI, confidence interval.

#### Risk factors for severe LRTIs

Multivariate Cox regression analysis demonstrated that a higher daily dose of prednisone (HR: 1.25; 95% CI: 1.02–1.53; P = 0.035) and the presence of HGG (HR: 7.50; 95% CI: 2.78–20.20; P < 0.001) were associated with a greater risk of the first severe lower respiratory tract infection (**Table 5**).

**Table 5:** Multivariate cox regression analysis of the first Severe Lower Respiratory Tract Infection.

Variables	No (n = 168)	Yes (n = 18)	HR (95% CI)	*P*
Prednisone daily dose/5 mg	4.00 (4.00, 6.00)	8.00 (4.00, 8.00)	1.25 (1.02 ~ 1.53)	0.035
HGG (time-dependent covariate)	42 (25.00)	9 (50.00)	7.50 (2.78 ~ 20.20)	<0.001

HGG, hypogammaglobulinemia; HR, hazard ratio; CI, confidence interval.

#### Risk factors for HGG

Multivariate Cox regression analysis revealed that compared with patients without bulbar or respiratory involvement at baseline, patients with isolated bulbar involvement had a significantly lower risk of developing HGG (HR: 0.46; 95% CI: 0.22–0.97; P = 0.040) (**Table 6**). No other factors were found to be independently associated with the development of HGG.

**Table 6:** Multivariate cox regression analysis of HGG.

Variables	No (n = 135)	Yes (n = 51)	HR (95% CI)	*P*
Age, years	49.00 (37.00, 62.00)	57.00 (41.50, 67.50)	1.01 (1.00 ~ 1.03)	0.121
Female	82 (60.74)	22 (43.14)	0.60 (0.34 ~ 1.07)	0.084
Bulbar/Respiratory involvement				
None	37 (27.41)	16 (31.37)	1.00 (Reference)	
Respiratory only	11 (8.15)	4 (7.84)	0.88 (0.29 ~ 2.65)	0.819
Bulbar only	58 (42.96)	13 (25.49)	0.46 (0.22 ~ 0.97)	0.040
Bulbar + Respiratory	29 (21.48)	18 (35.29)	1.26 (0.63 ~ 2.51)	0.511

HGG, hypogammaglobulinemia; HR, hazard ratio; CI, confidence interval.

### Sensitivity analysis

**Figure 4:**
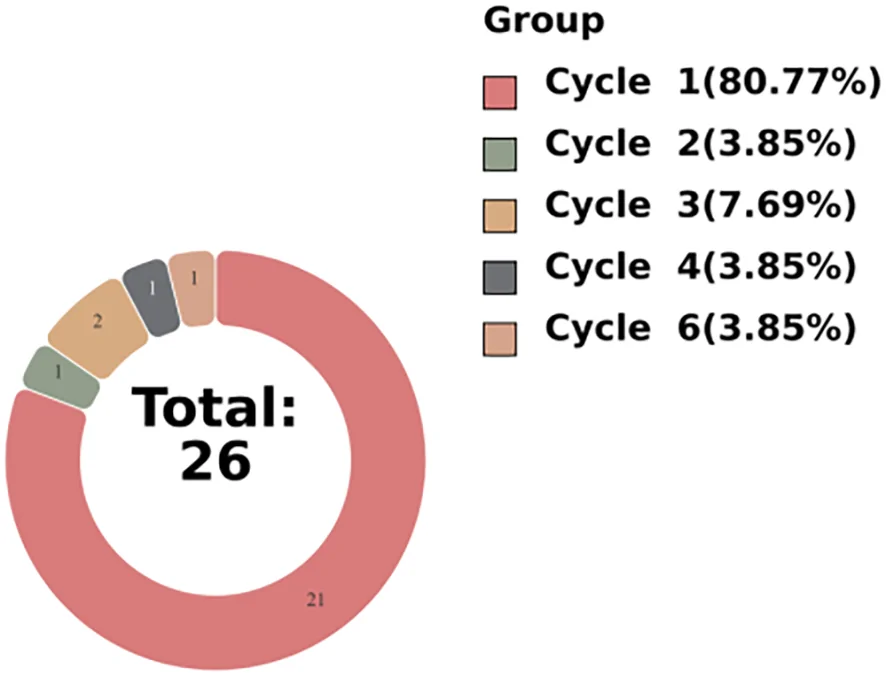
Treatment cycle at first infection after excluding three patients with COVID-19 infection.

After excluding 3 patients with coronavirus disease 2019 (COVID-19)-related infections, 26 patients (14.21%) were found to contract infections. The infection incidence rates at 1 and 2 years after the initiation of RTX treatment were 12.32 (95% CI, 7.49–19.00) and 8.60 (95% CI, 5.57–12.65) per 100 person-years, respectively, with 21 patients (80.77%) occurring during the first RTX treatment cycle (**Figure 4**). Subsequent risk factor analysis showed that in multivariate Cox regression, HGG remained a strong independent risk factor for non-COVID-19 infections (HR: 3.33; 95% CI: 1.49–7.43; P = 0.003). Additionally, each 5-mg increase in the daily dose of prednisone was associated with a 17% increase in the risk of infection (HR: 1.17; 95% CI: 0.98–1.40; P = 0.081) (**Supplementary Table 3**).

Following exclusion of the same 3 COVID-19–related cases, severe LRTIs occurred in 15 patients (8.20%). The incidence rates at one and two years were 6.78 (95% CI, 3.40–12.17) and 5.16 (95% CI, 2.89–8.53) per 100 person-years, respectively. Higher daily doses of prednisone (HR: 1.34; 95% CI: 1.06–1.69; P = 0.014) and fewer RTX treatment cycles (HR: 0.63; 95% CI: 0.42–0.93; P = 0.019) had significant statistical associations with higher severe LRTI risk in univariate analysis (**Supplementary Table 4**).

HGG was categorized into four subgroups: no HGG (reference group), isolated IgG deficiency, isolated IgM deficiency, and other subtypes (including combined deficiency of multiple immunoglobulins or isolated IgA deficiency). We further analyzed the impacts of these HGG subtypes on overall infections and severe LRTIs. Multivariate analyses demonstrated that compared with patients without HGG, isolated IgM deficiency (HR: 6.93, 95% CI: 2.63–18.30, P<0.001) and other HGG subtypes (HR: 14.20, 95% CI: 3.24–62.40, P<0.001) were significantly associated with elevated infection risk (**Supplementary Table 5**). For severe LRTIs, isolated IgM deficiency (HR: 7.82, 95%CI: 2.39–25.50, P<0.001) and other subtypes (HR: 25.40, 95%CI: 5.36–120.00, P<0.001) also conferred significantly higher risk relative to the reference group. In addition, each 5-mg increment in daily baseline prednisone dosage corresponded to a 24% increase in the risk of severe LRTIs (HR: 1.24, 95% CI: 1.01–1.53, P = 0.040) (**Supplementary Table 6**).

## Discussion

Researchers have extensively investigated the pleiotropic effects of RTX on the immune system in recent years. However, data on infection-related outcomes in patients with MG receiving RTX are limited, particularly in the context of low-dose regimens, where systematic real-world evidence is scarce. Existing safety assessments are largely derived from subgroup analyses of randomized controlled trials, which may not fully capture the complexity of the risk of infection encountered in routine clinical practice. In this study, we comprehensively characterized infection events and hypogammaglobulinemia in MG patients treated with low-dose RTX in a real-world setting. We also identified key risk factors associated with these adverse outcomes, providing clinically relevant evidence to inform the safe use and monitoring of RTX in the management of MG.

In our clinical cohort, treatment with low-dose RTX (500 mg every six months) was associated with infection incidence rates of 13.41 (95% CI, 8.47-20.28) and 9.20 (95% CI, 6.06-13.36) per 100 person-years at 1 and 2 years after the initiation of treatment, respectively, with an overall infection rate of 15.59%. This incidence was substantially lower than the total infection rate (48.3%) reported in other studies (19). This difference may be explained by two main factors. First, our study followed a low-dose RTX regimen, whereas most studies used standard-dose protocols, such as induction with 1000 mg of RTX administered twice two weeks apart, followed by the infusion of 1000 mg of RTX every six months for maintenance (10), or the conventional regimen of 375 mg/m^2^ RTX every week for four consecutive weeks (12, 14). Second, all patients underwent rigorous pre-treatment screening for parameters that are associated with infections, including C-reactive protein, immunoglobulin levels, T-SPOT, and B-lymphocyte counts, all of which were required to be within normal reference ranges. This rigorous screening protocol may have reduced the overall infection rate. Although the baseline screening results were normal, two patients who had MG crisis complicated by pulmonary infection within 3 months before the initiation of RTX treatment, developed severe pneumonia following the first treatment cycle. This observation suggests that a recent severe infection may represent a latent risk factor for post-RTX infections. Therefore, caution should be maintained while prescribing RTX to MG patients with recent severe episodes of infection, following thorough individualized risk assessment (20).

Rituximab (RTX) exerts broad immunomodulatory effects through multiple mechanisms. It selectively depletes memory B cells, leading to HGG and late-onset neutropenia (21, 22). It also reshapes the interactions between B cells and T cells, thereby indirectly modulating the induction, maintenance, and activation of T-cell-mediated immune responses (23) or directly reducing the number of CD20^+^ T cells (24). In a study involving 15 patients with MG, RTX-induced depletion of B cells was accompanied by an increase in the proportion of regulatory T cells (Tregs) (25). This multidimensional depletion and imbalance of the immune system substantially increases susceptibility to infections. A study reported that RTX-associated infections encompass viral, bacterial, fungal, and parasitic pathogens (26, 27). Khalaf Kridinba reported a significant increase in the risk of COVID-19, parasitic infections, and cytomegalovirus (CMV) infection within the first 12 months following RTX treatment (26). Similar to these findings, our study demonstrated that following treatment with low-dose RTX, patients with MG mainly developed bacterial and fungal infections, followed by viral infections. Fungal pneumonia carries significant harm and often presents with atypical clinical features; in the early stage of the infection, patients may be afebrile with only subtle radiographic abnormalities on chest CT scans. Accordingly, heightened clinical vigilance is warranted to avoid delayed diagnosis and treatment.

In our cohort, RTX-induced HGG predominantly manifested as reductions in IgM and IgG levels, whereas the low IgA was relatively uncommon, consistent with previous reports (28). This immunoglobulin isotype-specific profile may reflect differential sensitivities of B-cell subsets to RTX. Splenic marginal zone B cells, the primary source of IgM, are highly susceptible to RTX-mediated depletion (29, 30). In contrast, IgA levels may be preserved through dual protective mechanisms: long-lived CD20^-^ plasma cells that evade RTX targeting and continue immunoglobulin secretion, as well as IgA^+^ plasmablasts in mucosal tissues that are resistant to RTX and act as an independent, sufficient source of IgA (31).

In multivariate Cox regression analyses, HGG was identified as an independent risk factor for infection, and this association remained significant for severe LRTIs. Given that severe LRTIs are closely associated with increased hospitalization rates, mortality, and exacerbation of MG (17, 32), this finding has important clinical implications. Consistent with our results, another study on patients with multiple sclerosis receiving RTX demonstrated that higher IgG levels were associated with a lower risk of infection (HR = 0.86, 95% CI: 0.75–0.98, P = 0.029) (10), highlighting the critical role of humoral immunity in susceptibility to infection. As patients with baseline hypogammaglobulinemia were excluded from our cohort, indicating that the *de novo* development of HGG during RTX treatment represents a key driver of the risk of infection. Collectively, these findings emphasize the importance of regular monitoring and dynamic assessment of immunoglobulin levels in MG patients treated with low-dose RTX.

Concomitant prednisone use was also closely associated with the risk of infection. It has been previously established that prednisone increase susceptibility to infections through broad suppression of innate and adaptive immune responses (33, 34). In our multivariate analysis, the daily dose of prednisone was associated with the risk of severe LRTIs. These results highlight that careful control of the dosage of prednisone, particularly early achievement of steroid tapering, is critical for preventing infection in MG patients receiving RTX. In the multivariate analysis of HGG onset, isolated bulbar involvement was statistically correlated with reduced HGG risk. This finding may arise from random error and represent a chance result; therefore, this association should not be overinterpreted.

This real-world retrospective cohort study provides clinically relevant insights into infection and the risk of HGG in MG patients treated with low-dose RTX. Along with previous evidence, our findings indicate that alterations in immunoglobulins and exposure to prednisone are key determinants of RTX-associated risk of infections (26, 35). Additional factors, including advanced age (36), a history of severe infections (20), baseline immune cell profile (37), and comorbidities (18), may further modulate susceptibility to infections. Genetic predisposition may also play a role-for example, non-Hodgkin lymphoma patients carrying the F allele at the FCGR3A-158 locus have a higher risk of bloodstream infections following RTX therapy (38). This study identified multiple high-risk factors for infection, indicating that timely targeted preventive interventions are essential for high-risk patients. Studies by Kronbichler et al. and Park et al. suggested that (39, 40), prophylactic trimethoprim-sulfamethoxazole (TMP/SMX) may be considered for MG patients receiving ≥30 mg/day of prednisone, those aged ≥60 years, or individuals with underlying structural lung disease. Although the risk of adverse events may increase after prophylactic therapy (40, 41), individualized risk assessment and targeted preventive strategies during the early phase of RTX treatment may improve overall safety profiles.

This study had several limitations. First, we conducted a single-center retrospective study with a relatively small sample size, and reliance on medical records may have introduced selection and information bias. Future multicenter prospective studies with larger cohort studies need to be conducted to validate our findings. Second, analyses in the present study were performed based on baseline doses of prednisone and immunosuppressants, which failed to accurately and comprehensively obtain information regarding individualized prednisone tapering regimens, medication modifications, and actual drug exposure throughout follow-up. Prospective cohort studies are warranted in future to further verify our findings with time-varying indicators of medication exposure. Third, vaccination status acts as a critical confounder that affects the incidence of respiratory tract infections, particularly pneumococcal pneumonia, influenza, and COVID-19. However, relevant data were not systematically collected in this study. Future studies should comprehensively document patients’ vaccination histories and adopt multivariate adjustment to improve the reliability of conclusions. Fourth, the absence of RTX-related genetic testing and detailed T-cell subset analyses limited mechanistic interpretation at the molecular-genetic and cellular-immunological levels. Future studies that incorporate these immunogenetic and cellular immune parameters may facilitate the development of more comprehensive risk prediction models.

## Conclusion

Among patients with MG who were administered long-term low-dose RTX maintenance therapy (500 mg every 6 months), the overall incidence of infection was relatively low, with LRTIs being the most common type of infection. New-onset HGG during follow-up was an independent risk factor for any infection and severe lower respiratory tract infection. While higher baseline prednisone doses were associated with severe LRTIs. These findings suggest that in clinical practice, close monitoring of serum immunoglobulin levels and rational adjustment of glucocorticoid doses are essential for improving the safety of RTX therapy. Individualized risk assessment, including consideration of infection history, age, and comorbidities, should be performed for all MG patients before and during RTX treatment, and targeted preventive strategies should be implemented for high-risk patients to reduce infection-related morbidity and mortality. This study provides real-world evidence on the safety profile of low-dose RTX in treating MG, and offers insights for optimizing strategies to prevent infections.

## Data Availability

The original contributions presented in the study are included in the article/**Supplementary Material**. Further inquiries can be directed to the corresponding author/s.
