# Supplementary material for: Infections and hypogammaglobulinemia in myasthenia gravis patients receiving low-dose rituximab: a real-world cohort study
Source: Front Immunol. 2026 Aug 28;17:1907574. doi: 10.3389/fimmu.2026.1907574 (PMC13561787; doi:10.3389/fimmu.2026.1907574)
Supplement: Supplementary file 1 [file Table1.docx]

**Supplementary Table**

**Table S1.** Baseline characteristics of the 29 Patients excluded.

| **Variables** | **Total (n = 29)** |
| --- | --- |
| **Demographic characteristics** |  |
| Age, Y, Median (IQR) | 41.00 (29.00, 55.00) |
| Female, n(%) | 16/29 (55.17) |
| Smoking history | 2/29 (6.90) |
| Alcohol history | 1/29 (3.45) |
| **MG disease characteristics** |  |
| Disease duration, Months, Median (IQR) | 18.00 (11.00, 99.00) |
| Antibody type, n(%) |  |
| AchR | 23 (79.31) |
| Musk | 3 (10.34) |
| other | 2 (6.90) |
| Seronegative | 1 (3.45) |
| MGFA stage, n(%) |  |
| I | 7 (24.14) |
| IIA | 3 (10.34) |
| IIB | 7 (24.14) |
| IIIA | 1 (3.45) |
| IIIB | 7 (24.14) |
| IVA | 0 (0.00) |
| IVB | 2 (6.90) |
| V | 2 (6.90) |
| MG-ADL, Median (IQR) | 6.00 (2.00, 8.00) |
| QMG, Median (IQR) | 14.00 (5.00, 20.00) |
| **Comorbidities** |  |
| Diabetes, n(%) | 4/29 (13.79) |
| Hypertension, n(%) | 5/29 (17.24) |
| CV/CVD, n(%) | 1/29 (3.45) |
| Thyroid disease, n(%) | 0/29 (0.00) |
| Other autoimmune disease, n(%) | 1/29 (3.45) |
| Bulbar/Respiratory involvement, n(%) |  |
| Bulbar only | 6/29 (20.69) |
| Respiratory only | 3/29 (10.34) |
| Bulbar + Respiratory | 9/29 (31.03) |
| **B cell counts** |  |
| CD19+/CD27+B, Median (IQR) | 3.12 (2.36, 3.47) |
| CD19+B, Median (IQR) | 8.71 (6.47, 12.54) |
| CD20+B, Median (IQR) | 8.46 (6.60, 12.88) |
| **RTX treatment cycles**, Median (Range) | 1.00 (1.00 - 9.00) |
| Follow-up time, Years, Median (IQR) | - |
| **Concomitant medications**, n(%) | 28/29 (96.55) |
| Pyridostigmine, n(%) | 26/29 (89.66) |
| Pyridostigmine daily dose, mg, Median (IQR) | 180.00 (180.00, 360.00) |
| Prednisone, n(%) | 22/29 (75.86) |
| Prednisone daily dose, mg, Median (IQR) | 20.00 (10.00, 30.00) |
| Non-steroidal immunosuppressant, n(%) | 14/29 (48.28) |
| **Type of non-steroidal immunosuppressant**, n(%) |  |
| Efgartigimod | 0 (0.00) |
| Azathioprine | 2 (6.90) |
| Mycophenolate mofetil | 0 (0.00) |
| Tacrolimus | 12 (41.38) |

Abbreviations: IQR, interquartile range; AChR, acetylcholine receptor; MuSK, muscle-specific receptor tyrosine kinase; MGFA, Myasthenia Gravis Foundation of America Clinical Classification; MG-ADL, Myasthenia Gravis Activities of Daily Living; QMG, Quantitative Myasthenia Gravis; CV/CVD, cardiovascular/cerebrovascular disease; RTX, rituximab; Range, observed range.

**Table S2**. Baseline characteristics, immunologic status, and infection details of the 29 Patients with infections.

| **ID** | **Sex** | **Age** | **MGFA** | **HGG** | **Prednisone**  **-Daily Dosage** | **Infection Cycle** | **Severity of Infection** | **Infection type** | **Infecting microorganism** | **Treatment** |
| --- | --- | --- | --- | --- | --- | --- | --- | --- | --- | --- |
| 1 | M | 56 | I | Yes | 10 | 1 | Grade 2 | URTI | ND | Amoxicillin / Traditional Chinese medicine |
| 2 | F | 64 | I | No | 30 | 6 | Grade 3 | Perianal abscess | ND | Piperacillin-tazobactam |
| 3 | M | 41 | IIIB | No | 40 | 1 | Grade 5 | Pneumonia | Acinetobacter baumannii / Staphylococcus haemolyticus | Cefoperazone-sulbactam |
| 4 | M | 84 | IIIA | Yes | 40 | 1 | Grade 1 | Pneumonia | Mycobacterium tuberculosis | Untreated |
| 5 | F | 58 | IIIB | No | 30 | 1 | Grade 4 | Cholecystitis | ND | Ceftriaxone + surgical intervention |
| 6 | F | 50 | IIB | No | 45 | 1 | Grade 4 | Pneumonia | Pneumocystis jirovecii | Cotrimoxazole |
| 7 | M | 74 | IIIB | Yes | 20 | 3 | Grade 4 | Pneumonia | SARS-CoV-2 | Azvudine |
| 8 | F | 54 | IVB | No | 40 | 1 | Grade 4 | Pneumonia / Perianal abscess | ND | Unknown |
| 9 | F | 37 | IIIA | No | 20 | 4 | Grade 2 | Skin herpes | Herpes simplex virus | Acyclovir |
| 10 | M | 62 | IIB | Yes | 50 | 5 | Grade 4 | Pneumonia | SARS-CoV-2 | Symptomatic treatment |
| 11 | M | 62 | IIB | Yes | 30 | 1 | Grade 3 | Pneumonia | Pneumocystis jirovecii | Cotrimoxazole |
| 12 | M | 36 | IIB | No | 45 | 1 | Grade 4 | Pneumonia | Klebsiella pneumoniae / Streptococcus pneumoniae / Mucor | Ceftazidime-avibactam / Voriconazole / Vancomycin |
| 13 | F | 28 | IIIA | Yes | 40 | 1 | Grade 4 | Encephalitis | Herpes simplex virus | Acyclovir |
| 14 | M | 57 | IIIA | Yes | 40 | 3 | Grade 2 | Pneumonia | ND | Cefditoren |
| 15 | M | 30 | IIB | No | 20 | 2 | Grade 4 | Pneumonia / UTI | SARS-CoV-2 / Pseudomonas aeruginosa | Azvudine / Cefditoren |
| 16 | F | 71 | IIB | No | 20 | 1 | Grade 5 | Pneumonia | Acinetobacter baumannii / Pneumocystis jirovecii | Cefoperazone-sulbactam / Imipenem / Caspofungin / Voriconazole |
| 17 | M | 58 | IIIB | No | 40 | 1 | Grade 5 | Pneumonia | ND | Cefoperazone-sulbactam |
| 18 | F | 57 | IIA | Yes | 16 | 1 | Grade 2 | URTI | ND | Oral amoxicillin / Traditional Chinese medicine |
| 19 | F | 25 | I | Yes | 8 | 1 | Grade 3 | Pneumonia | ND | Ceftazidime / Doxycycline |
| 20 | M | 61 | I | No | 16 | 1 | Grade 2 | Skin herpes | Herpes simplex virus | Acyclovir |
| 21 | F | 60 | IIIB | No | 40 | 1 | Grade 4 | Pneumonia | Pneumocystis jirovecii | Cotrimoxazole |
| 22 | M | 62 | IVB | Yes | 8 | 3 | Grade 3 | Pneumonia | ND | Cefixime |
| 23 | F | 67 | IIA | Yes | 25 | 2 | Grade 4 | Pneumonia | Streptococcus pneumoniae | Cefoperazone-sulbactam / Doxycycline |
| 24 | F | 35 | I | No | - | 1 | Grade 4 | Pneumonia | Pneumocystis jirovecii | Cotrimoxazole |
| 25 | M | 62 | IIA | No | - | 1 | Grade 3 | URTI | ND | Symptomatic treatment |
| 26 | F | 66 | IIA | Yes | 15 | 1 | Grade 2 | URTI | ND | Cefditoren |
| 27 | M | 48 | IIIB | Yes | 40 | 1 | Grade 2 | URTI | ND | Cefixime |
| 28 | F | 54 | I | Yes | 40 | 1 | Grade 4 | Pneumonia | Pneumocystis jirovecii | Sivelestat / Piperacillin-tazobactam / Cotrimoxazole |
| 29 | F | 52 | IIIB | Ye | 30 | 1 | Grade 4 | Pneumonia | Haemophilus influenzae / Pneumocystis jirovecii | Ceftazidime / Cotrimoxazole (SMX-TMP) |

Abbreviations**:** M: male; F: female; HGG: Hypogammaglobulinemia; UTI: Urinary tract infection；URTI: Upper respiratory tract infection; ND：The causative pathogens were unidentified. “Prednisone Daily Dosage” refers to the baseline dose; “-” indicates that the patient was not receiving glucocorticoid therapy.

**Table S3.** Cox regression analysis of the first infection after excluding 3 patients with COVID-19 infection

| Variables | Infectious events | |  |  | Cox Regression Analysis | | | |  |
| --- | --- | --- | --- | --- | --- | --- | --- | --- | --- |
|  | No(n = 157) | Yes (n = 26) |  |  | Univariate | |  | Multivariate | |
|  |  |  |  |  | HR (95%CI) | *P* |  | HR (95%CI) | *P* |
| Age, years | 50.00 (37.00, 64.00) | 57.00 (48.50, 62.00) |  |  | 1.01 (0.99 ~ 1.04) | 0.319 |  | |  |
| Female | 89 (56.69) | 15 (57.69) |  |  | 1.02 (0.47 ~ 2.23) | 0.955 |  | |  |
| Smoking history | 24 (15.29) | 5 (19.23) |  |  | 1.34 (0.51 ~ 3.55) | 0.557 |  | |  |
| Alcohol history | 10 (6.37) | 3 (11.54) |  |  | 1.90 (0.57 ~ 6.34) | 0.295 |  | |  |
| Disease duration, months | 26.00 (10.00, 65.00) | 17.50 (3.50, 51.25) |  |  | 1.00 (0.99 ~ 1.01) | 0.477 |  | |  |
| MG antibody type |  |  |  |  |  |  |  | |  |
| AchR | 113 (71.97) | 20 (76.92) |  |  | 1.00 (Reference) |  |  | |  |
| Musk | 23 (14.65) | 4 (15.38) |  |  | 0.93 (0.32 ~ 2.71) | 0.889 |  | |  |
| Other | 4 (2.55) | 0 (0.00) |  |  | 0.00 (0.00 ~ Inf) | 0.997 |  | |  |
| Seronegative | 17 (10.83) | 2 (7.69) |  |  | 0.76 (0.18 ~ 3.28) | 0.718 |  | |  |
| MGFA stage |  |  |  |  |  |  |  | |  |
| I | 29 (18.47) | 6 (23.08) |  |  | 1.00 (Reference) |  |  | |  |
| II | 83 (52.87) | 8 (30.77) |  |  | 0.45 (0.16 ~ 1.31) | 0.144 |  | |  |
| III | 35 (22.29) | 10 (38.46) |  |  | 1.27 (0.46 ~ 3.51) | 0.638 |  | |  |
| IV or higher | 10 (6.37) | 2 (7.69) |  |  | 0.83 (0.17 ~ 4.11) | 0.818 |  | |  |
| MG-ADL | 5.00 (3.00, 8.00) | 5.50 (3.25, 10.00) |  |  | 1.06 (0.96 ~ 1.16) | 0.254 |  | |  |
| QMG | 12.00 (7.00, 17.00) | 13.00 (7.25, 19.75) |  |  | 1.02 (0.96 ~ 1.08) | 0.579 |  | |  |
| Diabetes mellitus | 22 (14.01) | 4 (15.38) |  |  | 1.06 (0.37 ~ 3.08) | 0.911 |  | |  |
| Hypertension | 40 (25.48) | 10 (38.46) |  |  | 1.66 (0.75 ~ 3.66) | 0.208 |  | |  |
| CV/CVD | 4 (2.55) | 2 (7.69) |  |  | 2.93 (0.69 ~ 12.39) | 0.145 |  | |  |
| Thyroid disease | 7 (4.46) | 0 (0.00) |  |  | 0.00 (0.00 ~ Inf) | 0.997 |  | |  |
| Other autoimmune diseases | 5 (3.18) | 0 (0.00) |  |  | 0.00 (0.00 ~ Inf) | 0.998 |  | |  |
| Bulbar/Respiratory involvement |  |  |  |  |  |  |  | |  |
| None | 45 (28.66) | 8 (30.77) |  |  | 1.00 (Reference) |  |  | |  |
| Respiratory only | 12 (7.64) | 3 (11.54) |  |  | 1.43 (0.38 ~ 5.40) | 0.596 |  | |  |
| Bulbar only | 64 (40.76) | 6 (23.08) |  |  | 0.49 (0.17 ~ 1.42) | 0.188 |  | |  |
| Bulbar + Respiratory | 36 (22.93) | 9 (34.62) |  |  | 1.24 (0.48 ~ 3.23) | 0.653 |  | |  |
| Pyridostigmine | 151 (96.18) | 24 (92.31) |  |  | 0.46 (0.11 ~ 1.96) | 0.297 |  | |  |
| Pyridostigmine daily dose, mg | 240.00 (180.00, 345.00) | 180.00 (180.00, 292.50) |  |  | 1.00 (0.99 ~ 1.00) | 0.592 |  | |  |
| Prednisone | 131 (83.44) | 24 (92.31) |  |  | 2.09 (0.49 ~ 8.84) | 0.317 |  | |  |
| Prednisone daily dose/5 mg | 4.00 (4.00, 6.00) | 6.00 (3.80, 8.00) |  |  | 1.20 (1.01 ~ 1.43) | 0.036 | 1.17 (0.98~ 1.40) | | 0.081 |
| Non-steroidal immunosuppressants | 47 (29.94) | 7 (26.92) |  |  | 0.95 (0.40 ~ 2.25) | 0.901 |  | |  |
| CD19+/CD27+B | 2.91 (2.00, 5.11) | 2.61 (1.77, 5.79) |  |  | 1.03 (0.89 ~ 1.20) | 0.658 |  | |  |
| CD20+B | 10.33 (7.07, 15.29) | 12.60 (8.32, 15.31) |  |  | 1.00 (0.94 ~ 1.06) | 0.977 |  | |  |
| CD21+B | 10.16 (6.52, 15.21) | 12.54 (8.44, 14.85) |  |  | 1.00 (0.94 ~ 1.06) | 0.913 |  | |  |
| RTX treatment cycles | 3.00 (2.00, 4.00) | 1.00 (1.00, 4.00) |  |  | 0.89 (0.71 ~ 1.11) | 0.291 |  | |  |
| HGG (time-dependent covariate) | 36 (22.93) | 13 (50.00) |  |  | 4.87 (2.06 ~ 11.50) | <0.001 | 4.73 (1.97 ~ 11.30) | | <0.001 |

Abbreviations: MG, myasthenia gravis; MGFA, Myasthenia Gravis Foundation of America; MG-ADL, Myasthenia Gravis Activities of Daily Living; QMG, Quantitative Myasthenia Gravis; CV/CVD, cardiovascular/cerebrovascular disease; CD, cluster of differentiation; RTX, rituximab; HGG, hypogammaglobulinemia; HR, hazard ratio; CI, confidence interval.

**Table S4.** Cox regression analysis of the first severe lower respiratory tract infection after excluding 3 patients with COVID-19 infection

| Variables | Infectious events | |  | Univariate | | |
| --- | --- | --- | --- | --- | --- | --- |
|  |  |  |  |  |  |  |
|  | No(n = 168) | Yes (n = 15) |  |  | HR (95%CI) | *P* |
| Age, years | 51.00 (37.00, 64.00) | 54.00 (45.50, 61.00) |  |  | 1.01 (0.98 ~ 1.04) | 0.733 |
| Female | 95 (56.55) | 9 (60.00) |  |  | 1.12 (0.40 ~ 3.15) | 0.826 |
| Smoking history | 27 (16.07) | 2 (13.33) |  |  | 0.86 (0.19 ~ 3.81) | 0.841 |
| Alcohol history | 11 (6.55) | 2 (13.33) |  |  | 2.20 (0.50 ~ 9.76) | 0.299 |
| Disease duration, months | 26.00 (10.00, 64.25) | 11.00 (2.50, 21.50) |  |  | 0.99 (0.98 ~ 1.01) | 0.370 |
| MG antibody type |  |  |  |  |  |  |
| AchR | 124 (73.81) | 9 (60.00) |  |  | 1.00 (Reference) |  |
| Musk | 23 (13.69) | 4 (26.67) |  |  | 2.06 (0.64 ~ 6.71) | 0.228 |
| Other | 4 (2.38) | 0 (0.00) |  |  | 0.00 (0.00 ~ Inf) | 0.998 |
| Seronegative | 17 (10.12) | 2 (13.33) |  |  | 1.69 (0.37 ~ 7.87) | 0.500 |
| MGFA stage |  |  |  |  |  |  |
| I | 32 (19.05) | 3 (20.00) |  |  | 1.00 (Reference) |  |
| II | 86 (51.19) | 5 (33.33) |  |  | 0.57 (0.14 ~ 2.38) | 0.439 |
| III | 40 (23.81) | 5 (33.33) |  |  | 1.28 (0.30 ~ 5.34) | 0.739 |
| IV or higher | 10 (5.95) | 2 (13.33) |  |  | 1.62 (0.27 ~ 9.74) | 0.596 |
| MG-ADL | 5.00 (3.00, 8.00) | 7.00 (4.00, 10.00) |  |  | 1.09 (0.96 ~ 1.23) | 0.189 |
| QMG | 12.00 (7.00, 17.00) | 13.00 (8.50, 20.50) |  |  | 1.04 (0.96 ~ 1.12) | 0.371 |
| Diabetes mellitus | 23 (13.69) | 3 (20.00) |  |  | 1.45 (0.41 ~ 5.16) | 0.561 |
| Hypertension | 44 (26.19) | 6 (40.00) |  |  | 1.74 (0.62 ~ 4.89) | 0.293 |
| CV/CVD | 6 (3.57) | 0 (0.00) |  |  | 0.00 (0.00 ~ Inf) | 0.998 |
| Thyroid disease | 7 (4.17) | 0 (0.00) |  |  | 0.00 (0.00 ~ Inf) | 0.998 |
| Other autoimmune diseases | 5 (2.98) | 0 (0.00) |  |  | 0.00 (0.00 ~ Inf) | 0.998 |
| Bulbar/Respiratory involvement |  |  |  |  |  |  |
| None | 48 (28.57) | 5 (33.33) |  |  | 1.00 (Reference) |  |
| Respiratory only | 15 (8.93) | 0 (0.00) |  |  | 0.00 (0.00 ~ Inf) | 0.997 |
| Bulbar only | 67 (39.88) | 3 (20.00) |  |  | 0.40 (0.09 ~ 1.66) | 0.206 |
| Bulbar + Respiratory | 38 (22.62) | 7 (46.67) |  |  | 1.56 (0.49 ~ 4.92) | 0.450 |
| Pyridostigmine | 162 (96.43) | 13 (86.67) |  |  | 0.25 (0.06 ~ 1.12) | 0.070 |
| Pyridostigmine daily dose, mg | 240.00 (180.00, 352.50) | 180.00 (180.00, 270.00) |  |  | 1.00 (0.99 ~ 1.00) | 0.429 |
| Prednisone | 141 (83.93) | 14 (93.33) |  |  | 2.43 (0.32 ~ 18.49) | 0.391 |
| Prednisone daily dose/5 mg | 4.00 (4.00, 6.00) | 8.00 (5.25, 8.00) |  |  | 1.34 (1.06 ~ 1.69) | 0.014 |
| Non-steroidal immunosuppressants | 50 (29.76) | 4 (26.67) |  |  | 0.94 (0.30 ~ 2.95) | 0.912 |
| CD19+/CD27+B | 3.01 (2.00, 5.49) | 2.27 (1.72, 2.80) |  |  | 0.85 (0.66 ~ 1.09) | 0.203 |
| CD20+B | 10.59 (7.27, 15.24) | 9.53 (5.30, 15.44) |  |  | 0.97 (0.89 ~ 1.06) | 0.503 |
| CD21+B | 10.32 (7.05, 15.22) | 9.43 (5.29, 14.38) |  |  | 0.96 (0.88 ~ 1.05) | 0.423 |
| RTX treatment cycles | 3.00 (1.00, 4.00) | 1.00 (1.00, 2.50) |  |  | 0.63 (0.42 ~ 0.93) | 0.019 |
| HGG (time-dependent covariate) | 42 (25.00) | 7 (46.67) |  |  | 7.50 (2.55 ~ 22.00) | <0.001 |

Abbreviations: MG, myasthenia gravis; MGFA, Myasthenia Gravis Foundation of America; MG-ADL, Myasthenia Gravis Activities of Daily Living; QMG, Quantitative Myasthenia Gravis; CV/CVD, cardiovascular/cerebrovascular disease; CD, cluster of differentiation; RTX, rituximab; HGG, hypogammaglobulinemia; HR, hazard ratio; CI, confidence interval.

**Table S5.** Multivariate cox regression analysis of the first infection

| Variables | No(n = 157) | Yes (n = 29) | HR (95%CI) | *P* |
| --- | --- | --- | --- | --- |
| CV/CVD | 4 (2.55) | 3 (10.34) | 2.78 (0.78 ~ 9.96) | 0.099 |
| Prednisone daily dose/5 mg | 4.00 (4.00, 6.00) | 6.00 (4.00, 8.00) | 1.11 (0.94 ~ 1.31) | 0.175 |
| HGG (time-dependent covariate) |  |  |  |  |
| No | 121 (77.07) | 14 (48.28) | 1.00 (Reference) |  |
| isolated IgG deficiency | 10 (6.37) | 2 (6.90) | 1.60 (0.21 ~ 12.10) | 0.651 |
| isolated IgM deficiency | 20 (12.74) | 9 (31.03) | 6.93 (2.63 ~ 18.30) | <0.001 |
| Others | 6 (3.82) | 4 (13.79) | 14.20 (3.24 ~ 62.40) | <0.001 |

Abbreviations: CV/CVD, cardiovascular/cerebrovascular disease; HGG, hypogammaglobulinemia; HR, hazard ratio; CI, confidence interval.

Others: patients with combined deficiency of two or more immunoglobulin subtypes or isolated IgA deficiency.

**Table S6.** Multivariate cox regression analysis of the first Severe Lower Respiratory Tract Infection

| Variables | No(n = 168) | Yes (n = 18) | HR (95%CI) | *P* |
| --- | --- | --- | --- | --- |
| Prednisone daily dose/5 mg | 4.00 (4.00, 6.00) | 8.00 (4.00, 8.00) | 1.24 (1.01 ~ 1.53) | 0.040 |
| HGG (time-dependent covariate) |  |  |  |  |
| No | 126 (75.00) | 9 (50.00) | 1.00 (Reference) |  |
| isolated IgG deficiency | 10 (5.95) | 2 (11.11) | 2.83 (0.36 ~ 22.40) | 0.326 |
| isolated IgM deficiency | 24 (14.29) | 5 (27.78) | 7.82 (2.39 ~ 25.50) | <0.001 |
| Others | 8 (4.76) | 2 (11.11) | 25.40 (5.36 ~ 120.00) | <0.001 |

Abbreviations: HGG, hypogammaglobulinemia; HR, hazard ratio; CI, confidence interval. Others: patients with combined deficiency of two or more immunoglobulin subtypes or isolated IgA deficiency.
